# Newborn Screening for Long-Chain 3-Hydroxyacyl-CoA Dehydrogenase and Mitochondrial Trifunctional Protein Deficiencies Using Acylcarnitines Measurement in Dried Blood Spots—A Systematic Review of Test Accuracy

**DOI:** 10.3389/fped.2021.606194

**Published:** 2021-03-19

**Authors:** Chris Stinton, Hannah Fraser, Julia Geppert, Rebecca Johnson, Martin Connock, Samantha Johnson, Aileen Clarke, Sian Taylor-Phillips

**Affiliations:** ^1^Warwick Medical School, University of Warwick, Coventry, United Kingdom; ^2^School of Nursing, Midwifery and Health, Coventry University, Coventry, United Kingdom; ^3^Warwick Library, University of Warwick, Coventry, United Kingdom

**Keywords:** systematic review, test accuracy, LCHAD deficiency, MTP deficiency, newborn blood spot screening

## Abstract

**Background:** Long-chain 3-hydroxyacyl-CoA dehydrogenase (LCHAD) and mitochondrial trifunctional protein (MTP) deficiencies are rare autosomal recessive fatty acid β-oxidation disorders. Their clinical presentations are variable, and premature death is common. They are included in newborn blood spot screening programs in many countries around the world. The current process of screening, through the measurement of acylcarnitines (a metabolic by-product) in dried blood spots with tandem mass spectrometry, is subject to uncertainty regarding test accuracy.

**Methods:** We conducted a systematic review of literature published up to 19th June 2018. We included studies that investigated newborn screening for LCHAD or MTP deficiencies by tandem mass spectrometry of acylcarnitines in dried blood spots. The reference standards were urine organic acids, blood acylcarnitine profiles, enzyme analysis in cultured fibroblasts or lymphocytes, mutation analysis, or at least 10-year follow-up. The outcomes of interest were sensitivity, specificity, positive predictive value (PPV), and negative predictive value (NPV). Assessment of titles, abstracts, and full-text papers and quality appraisal were carried out independently by two reviewers. One reviewer extracted study data. This was checked by a second reviewer.

**Results:** Ten studies provided data on test accuracy. LCHAD or MTP deficiencies were identified in 23 babies. No cases of LCHAD/MTP deficiencies were identified in four studies. PPV ranged from 0% (zero true positives and 28 false positives from 276,565 babies screened) to 100% (13 true positives and zero false positives from 2,037,824 babies screened). Sensitivity, specificity, and NPV could not be calculated as there was no systematic follow-up of babies who screened negative.

**Conclusions:** Test accuracy estimates of screening for LCHAD and MTP deficiencies with tandem mass spectrometry measurement of acylcarnitines in dried blood were variable in terms of PPVs. Screening methods (including markers and thresholds) varied between studies, and sensitivity, specificity, and NPVs are unknown.

## Introduction

Long-chain 3-hydroxyacyl-CoA dehydrogenase (LCHAD) and mitochondrial trifunctional protein (MTP) deficiencies (Enzyme Commission Number 1.1.1.211) are recessive autosomal fatty acid β-oxidation disorders. They are caused by mutations in the genes coding for MTP. LCHAD deficiency arises as a result of mutations in the HADHA gene; MTP deficiency arises from mutations in HADHA and HADHB genes (1). The conditions are characterized by lethargy, hypoglycemia, hypotonia, cardiomyopathy, and acute metabolic crisis (2, 3). Long-term complications include liver disease, peripheral neuropathy, and retinopathy (3, 4). Signs and symptoms may present immediately after birth or later in life (5). Three main forms of LCHAD/MTP deficiencies have been reported: an early-onset form, which is associated with cardiomyopathy, hypoglycemia, and sudden infant death; an infant-onset form, which is characterized by recurrent hypoketotic hypoglycemia and lethargy during illness or fasting; and a milder, late-onset form that is triggered by exercise, fasting, or infections and is associated with progressive peripheral neuropathy and recurrent rhabdomyolysis (6, 7). There is no cure for LCHAD or MTP deficiencies, and premature death is common. Approximately 38% of infants die before, or within 3 months of, diagnosis (5). A number of management strategies are available, namely a high-carbohydrate and fat-modified/decreased diet that is low in long-chain fatty acids, supplements (l-carnitine, docosahexaenoic acid, and medium-chain triglyceride oil, such as triheptanoin), and avoidance of fasting (1). There is some evidence that these treatments are associated with improved clinical outcomes (e.g., reduced mortality, delayed visual complications), but the effects are variable, study sample sizes are small, and few data are available from long-term follow-up studies (1, 8–10). The incidence of LCHAD and MTP deficiencies varies widely around the world. A recent estimate from the USA gives an incidence of 1:363,738 for LCHAD deficiency and 1:1,240,467 for MTP deficiency (11).

It has been proposed that earlier recognition and treatment of LCHAD/MTP deficiencies may be critical for improving health outcomes (5), and the two conditions are included in newborn screening programs in many countries. Screening is conducted through the measurement of acylcarnitines (primarily C16OH, C16:1 OH, C18OH) in dried blood spots using tandem mass spectrometry (TMS). These markers are not specific to LCHAD/MTP deficiencies, however, and levels can be raised in other conditions (e.g., carnitine palmitoyltransferase II deficiency, very long-chain acyl-CoA dehydrogenase deficiency) and in babies of very low birth weight/being treated in neonatal intensive care (12). The results of a recent report from the UK suggest that screening for LCHAD/MTP deficiencies would not lead to the identification of additional cases as compared with the current practice of clinical detection (13). In contrast, data from other countries have suggested that screening does lead to earlier detection of LCHAD/MTP deficiencies (14, 15).

To date, there has been one systematic review examining test accuracy of screening for LCHAD/MTP deficiencies (16). Searching up to 2012, Einoder-Moreno et al. (16) identified six studies and concluded that sensitivity, specificity, and negative predictive value (NPV) of acylcarnitine measurement in dried blood spots are close to 100%, and that the positive predictive value (PPV) ranges from 9 to 100%. However, three relevant papers were missed by their search (12, 17, 18), and the calculation of sensitivity, specificity, and negative predictive value were based on an assumption about the disease status of babies who screened negative, as no follow-up of these babies was conducted in the included studies. This approach can lead to overestimation of sensitivity and underestimation of specificity (19). The aim of the current paper, therefore, is to conduct a systematic review of test accuracy metrics (sensitivity, specificity, positive and negative predictive values) of acylcarnitine measurement in newborn screening dried blood spots (DBS) for LCHAD/MTP deficiencies using tandem mass spectrometry using a broader search than the previous review and taking into consideration whether or not babies who screen negative received follow-up assessment.

## Materials and Methods

The review protocol is registered at PROSPERO (registration number CRD42018094356).

### Search Strategy

We conducted a search of the following electronic databases: MEDLINE, MEDLINE In-Process, MEDLINE Daily, MEDLINE ePub Ahead of Print, the Cochrane Library, Web of Science, and Embase. Search terms (free text and subject headings) related to the disease area (e.g., “mitochondrial trifunctional protein,” “long-chain-3-hydroxyacyl-CoA dehydrogenase,” “fatty acid oxidation disorder”) and screening (e.g., “newborn screening,” “dried blood spot,” “tandem mass spectrometry”). Full details of the search are provided in Supplement 1. The reference lists of included articles and relevant systematic reviews were also examined. The search was conducted on 19th June 2018, with no restrictions on the publication date or language of articles.

### Eligibility Criteria

We included journal articles and reports that investigated newborn screening for LCHAD or MTP deficiencies by TMS analysis of acylcarnitines in dried blood spots. The reference standards were urine organic acids, blood acylcarnitine profiles, enzyme analysis in cultured fibroblasts or lymphocytes, mutation analysis, or at least 10-year follow-up. These could be on their own or in any combination. Appropriate study designs were cross-sectional test accuracy studies, case-control studies, and cohort studies. The outcomes of interest were sensitivity, specificity, PPV, and NPV (or sufficient data to allow us to calculate these). We excluded non-human studies, letters, editorials, communications, conference abstracts, gray literature, studies of fatty acid β-oxidation disorders where data for LCHAD/MTP deficiencies could not be separated from data for other conditions, studies with no extractable data, and studies where more than 10% of the study sample did not meet our inclusion criteria.

### Screening and Data Extraction

Titles, abstracts, and full-text papers were independently screened by two reviewers. Data extraction was conducted by a single reviewer and checked by a second reviewer. At each stage of the review, disagreements were resolved through discussion between the reviewers, with the involvement of a third reviewer if consensus could not be achieved.

### Quality Appraisal

Two reviewers independently assessed risk of study bias and applicability concerns using the Quality Assessment Tool for Diagnostic Accuracy Studies (QUADAS-2) (20), which was tailored to the research question. Tailoring comprised defining cut-offs for exclusions, identifying appropriate reference standards, selecting a suitable interval between index tests and reference standards, and producing guidance notes. Disagreements were resolved through discussion by the two reviewers, leading to a consensus on assessment of risk of bias and applicability concerns for all studies. The QUADAS-2 tool is presented in Supplement 2 and the guidance notes in Supplement 3.

### Data Summary and Synthesis

Due to incomplete 2 × 2 tables and heterogeneity between study designs, a narrative summary of the evidence is provided. We calculated confidence intervals for test accuracy metrics using the Wilson score method with continuity correction (21).

## Results

### Searching, Sifting, and Sorting

**Figure 1:**
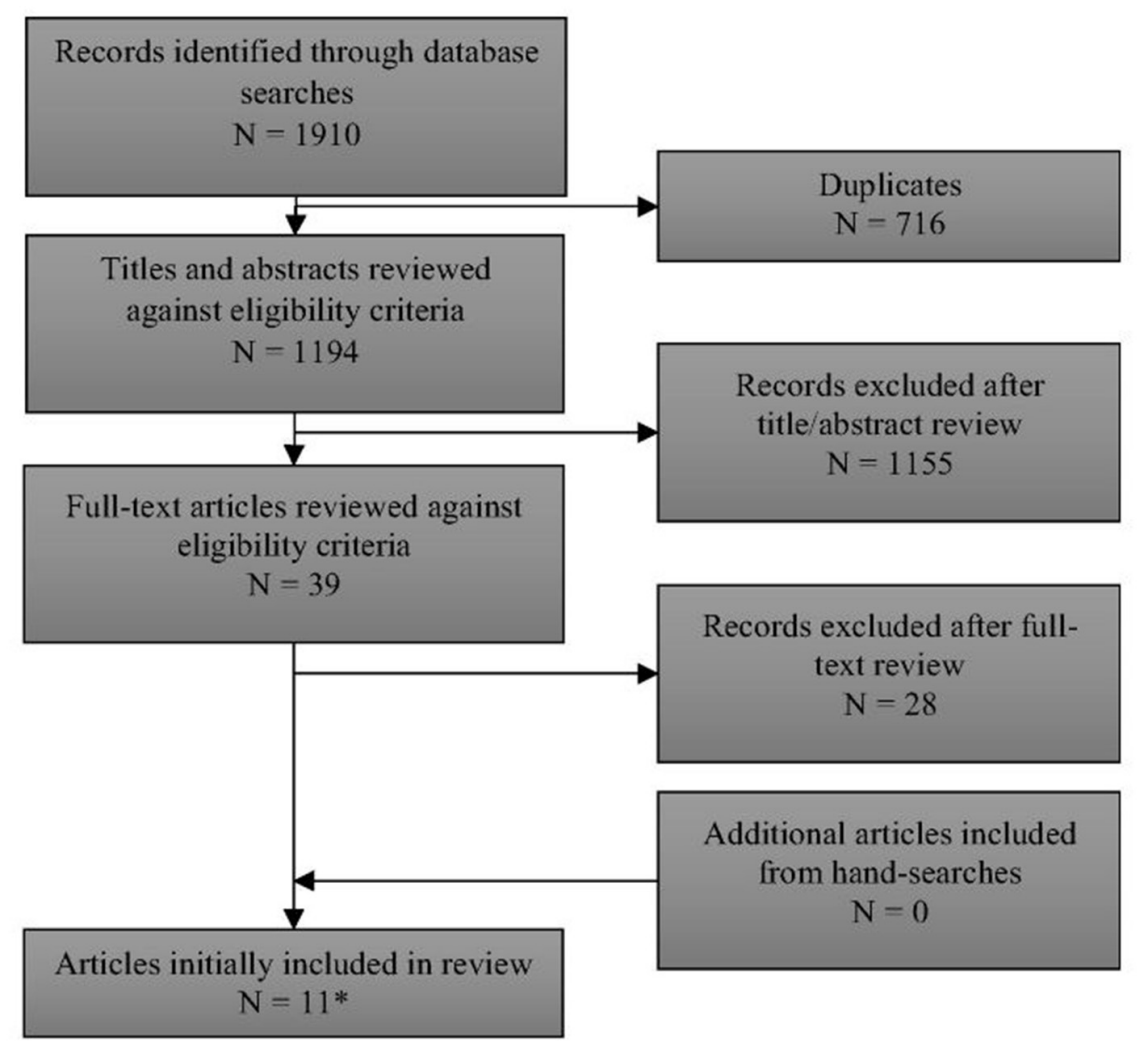
PRISMA flow diagram of records through the systematic review. *See Supplement 4 for list of excluded studies with reasons.

Full details of the flow of studies through the review are outlined in Figure 1. One thousand one hundred and ninety-four unique records were identified through searching electronic databases. After examination of titles and abstracts, 39 papers were retained for full-text assessment. Eleven of these papers met the review's inclusion criteria (12–15, 17, 18, 22–26). Two papers included overlapping cohorts (17, 22). Only the data from the larger, more recent paper by Lindner et al. (17) [which included all of the data from Schulze et al. (22)] are reported here. A list of excluded studies [with reasons for exclusion] is provided in Supplement 4.

### Quality Appraisal

**Figure 2:**
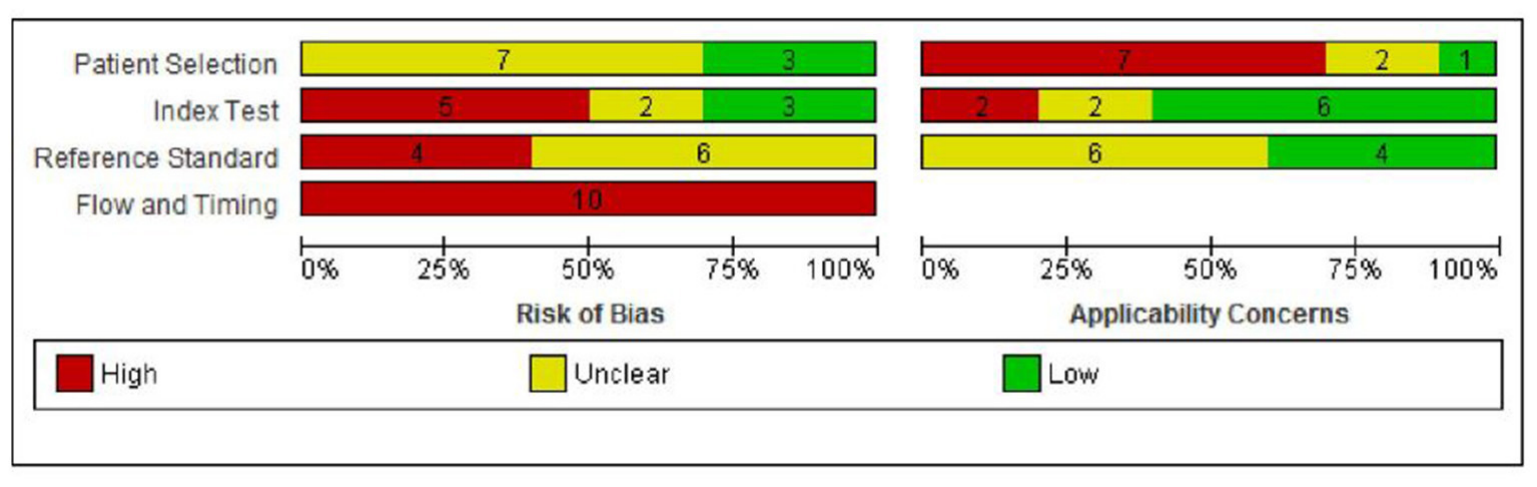
Risk of bias and applicability concern graph: review authors' judgments about each domain presented as percentages across included studies.

A summary of the risks of bias and applicability concerns of the included papers is provided in Figure 2. Ratings of risks of biases and applicability concerns for each individual study are provided in Supplement 5.

Risk of bias was considered to be high in two or more domains for nine of the 10 studies (90%) (12–14, 17, 18, 23–26) and in one domain for the remaining study (10%) (15). In the patient selection domain, risk of bias was rated as unclear in seven (70%) studies due to unclear/incomplete reporting (12, 15, 17, 23–26), and low in three (30%) studies (13, 14, 18). There were considerable concerns regarding applicability to the UK context in seven (70%) studies as blood samples were taken before day 5 in six studies (12, 15, 17, 18, 23, 25), and the incidence of MTPD was lower than expected in one study (1:300,000 compared with 1:149,254 in the UK) (24). Applicability concerns were unclear in two studies (14, 26) and low in one study (13).

In the index test domain, five studies (50%) were rated as having high risk of bias as the cut-off for “screen positive” was altered during the study period (13) or was not pre-specified (12, 23, 24, 26). Of the remaining studies, two were at unclear risk of bias (18, 25) and three were at low risk of bias (14, 15, 17). Two (20%) studies had high applicability concerns as one included additional markers (C14:1, C14-OH) (15) and one included both blood and urine samples (18). Applicability concerns were unclear in two studies (17, 25) and low in six studies (12–14, 23, 24, 26).

In the reference standard domain, risk of bias was rated as unclear in all 10 studies because it was not possible to tell the reference standard results were interpreted without knowledge of the results of the index test (12–15, 17, 18, 23–26). Applicability concerns were unclear in six (60%) studies (12, 15, 23–26) and low in the remaining four (40%) (13, 14, 17, 18).

Finally, all studies were judged to be at high risk of bias in the flow and timing domain (12–15, 17, 18, 23–26). The reasons for this were that the reference standards used to confirm disease status for screen positives and screen negatives were not the same, follow-up of those children who screened negative was not defined or not conducted, and losses to follow-up were not reported.

### Characteristics of Included Studies

Details of the included studies are provided in Table 1 and Supplement 6. Ten studies were included, four pilot programs (12, 13, 23, 25), three mixed pilot and national/regional screening programs (14, 17, 24), and three national/regional screening programs (15, 18, 26). Six studies took place in Europe [Denmark, Faroe Islands, and Greenland (14), Germany (15, 17), Slovenia (23), Spain (18), UK (13)], two in Asia (China (26), Hong Kong (25)), and two in North America [USA (12, 24)]. Sample sizes ranged from 2,440 (25) to 1,200,000 (15). In total, the 10 studies screened 3,951,358 newborns. Twenty-three true positives (TP) and 40 false positives (FP) were identified. The 23 true positives comprised 11 babies with LCHAD deficiency, two with MTP deficiency, and 10 babies for whom LCHAD and MTP deficiencies were not differentiated.

**Table 1:** Accuracy of newborn screening tests for LCHADD/MTPD using TMS measurement of acylcarnitines.

**References**	**Number screened**	**Marker(s)**	**Cut-off(s) (μmol/L)**	**2** **×** **2 table**				**Test accuracy**^****a****^			
				**TP**	**FP**	**FN**	**TN**	**Sensitivity** **(95% CI)**	**Specificity** **(95% CI)**	**PPV** (95% CI)*	**NPV** **(95% CI)**
Couce et al. (18)	210,165	C16OH, C18:1OH, C18OH	NR	2^b^	0	NR	NR	NA	NA	1 (0.20, 1)	NA
Frazier et al. (24)	239,415	C16OH, C18:1, C18:1OH	>0.18, >4.08, >0.14	2^b^	0	NR	NR	NA	NA	1 (0.20, 1)	NA
Lindner et al. (17)	1,084,195	C14OH (and/or): C16:1OH; C16OH; C18:1OH; C18OH	>0.12, >0.22, >0.20, >0.12, >0.11	6 (5 NBS, 1 cascade testing)^b^	0	NR	NR	NA	NA	1 (0.52, 1)	NA
Lund et al. (14)	504,049	Primary: C16OH; secondary: C18:1OH	>0.12U, >0.1U	3^c^	0	NR	NR	NA	NA	1 (0.31, 1)	NA
Mak et al. (25)	2,440	Unclear	Unclear	0	2	NR	NR	NA	NA	0 (0, 0.80)	NA
Sander et al. (15)	1,200,000	C16OH, C18:1OH, C14:1, C14OH	>0.08, >0.06, >0.35, >0.2	9 (7 LCHADD, 2 MTPD)	10	NR	NR	NA	NA	0.47 (0.25, 0.71)	NA
Smon et al. (23)	10,048	C16:1OH, C16OH, C18:1OH, C18 OH, C16OH/C16	0.042^d^ , 0.028^d^, 0.014^d^, 0.009^d^, 0.015^d^	0	8	NR	NR	NA	NA	0 (0, 0.40)	NA
UK NSC (13)	436,969	Primary: C16OH; secondary: C16-OH; C16:1-OH; C18-OH	>0.12, >0.15–lowered from 0.2	1^c^	2	NR	NR	NA	NA	0.33 (0.02, 0.87)	NA
Yang et al. (26)	100,077	LCHADD: C16OH; MTPD: C18OH, C18:1OH	>0.04, >0.03, >0.05	0	13 (4 LCHADD, 9 MTPD)	NR	NR	NA	NA	0 (0, 0.28)	NA
Zytkovicz et al. (12)	164,000	C16OH/d-C16	0.1^e^	0	5	NR	NR	NA	NA	0 (0, 0.54)	NA

a Confidence intervals calculated using Wilson score method with continuity correction.

b LCHAD deficiency.

c LCHAD/MTP deficiencies.

d Data provided by study authors.

e Determined during the study to ensure that no more than 0.02% of population would be flagged. CI, confidence interval; FN, false negative; FP, false positive; LCHADD, long-chain 3-hydroxyacyl-CoA dehydrogenase deficiency; MTPD, mitochondrial trifunctional protein deficiency; NA, not applicable; NBS, newborn bloodspot; NPV, negative predictive value; NR, not reported; PPV, positive predictive value; TN, true negative; TP, true positive; UK NSC, UK National Screening Committee. *The values were calculated by the review authors.

### Description of Screening and Diagnostic Tests

Details of the screening methodology and diagnostic tests used in each study are provided in Supplement 6. In brief, eight different analytes or ratios were used as markers to screen for LCHAD/MTP deficiencies: 3-hydroxytetradecanoylcarnitine (C14OH), tetradecenoylcarnitine (C14:1), 3-hydroxypalmitoylcarnitine (C16OH), 3-hydroxypalmitoleylcarnitine (C16:1OH), 3-hydroxystearoylcarnitine (C18OH), 3-hydroxyoleoylcarnitine (C18:1OH), oleoylcarnitine (C18:1), and 3-hydroxypalmitoylcarnitine/palmitoylcarnitine (C16OH/C16), with each study employing a unique combination. The cut-offs used varied between studies (e.g., cut-offs ranged from >0.04 to >0.20 μmol/L for C16OH, >0.03 to >0.15 μmol/L for C18OH, and >0.05 to >0.14 μmol/L for C18:1OH), with no two studies using the same combination of markers and thresholds. Screening samples were collected between 24 h and day 37 of life (25, 26).

The reference standards used varied between and within studies. For screen-positive babies, reference standards were blood acylcarnitines, urinary organic acids, and DNA analysis (13, 24, 25); enzyme and/or molecular studies (18); enzyme activity in fibroblasts/lymphocytes and mutation analysis (15); acylcarnitine profile in plasma/DBS and/or genotype and/or enzyme activity (17); urine organic acids, plasma acylcarnitines, and molecular-genetic analyses (14); organic acid in urine, next-generation sequencing, and an additional acylcarnitine profile in DBS (23); and urinary organic acids or DNA analysis (26). Lastly, one study used “standard metabolic criteria” (12). No systematic follow-up of babies who screened negative was conducted in any of the studies.

### Accuracy of Screening Tests

The cut-offs used to classify a positive case of LCHAD/MTP deficiencies and the diagnostic tests used to confirm this varied between studies. Therefore, we report positive screening results as those that met/exceeded the cut-off and were diagnostically confirmed as presented in the individual study. Test accuracy data are show in Table 1.

### Positive and Negative Predictive Values

PPV varied considerably between studies. It was 0% in four studies, with zero true positives and 28 false positives from 276,565 babies screened (12, 23, 25, 26), 33% in one study, with one true positive and two false positives from 436,969 babies screened (13), 47% in one study, with 9 true positives and 10 false positives from 1,200,000 babies screened (15), and 100% in four studies, with 13 true positives from 2,037,824 babies screened (14, 17, 18, 24). In the UK study, the single case reported as a true positive was being treated for LCHADD at the point of screening, as they had already been detected clinically (13). Confidence intervals were wide due to the small number of cases of LCHAD/MTP deficiencies detected (23 in total, zero to nine per study). It was not possible to calculate NPV as newborns who screened negative were not systematically followed up.

### Sensitivity and Specificity

We were not able to determine sensitivity or specificity due to a lack of information on babies who screened negative.

## Discussion

We assessed the test accuracy of acylcarnitine measurement in newborn DBS using TMS for LCHAD/MTP deficiencies. Ten relevant studies were identified. All studies had a high risk of bias in at least one domain, and 9/10 (90%) studies had a high risk of bias in at least two domains. Across the 10 studies, ~4,000,000 babies were screened and 23 cases of LCHAD/MTP deficiencies were identified; 11 babies had LCHAD deficiency, two had MTP deficiency, and 10 had undifferentiated LCHAD/MTP deficiencies. One of the cases reported as a true positive had already been detected clinically at the point at which screening took place (13). Arguably, the PPV for this study should be 0% rather than 33%, as reported in the study. Forty additional babies screened positive but were subsequently found not to have LCHAD or MTP deficiency. In four studies, no cases of LCHAD/MTP deficiencies were identified (12, 23, 25, 26). However, in three of these studies, the sample sizes were too small to be likely to detect such rare diseases [screening population sizes were 2,440 (25), 10,048 (23), and 100,077 (26)]. The fourth study included a larger sample (*n* = 164,000) but only included one marker (C16OH), which might have made the screening process less accurate (12).

The only measure of test accuracy that was consistently reported (or where sufficient data were present to allow us to calculate it) was PPV. PPV in the 10 studies ranged from 0% (zero true positives and 28 false positives from 276,565 babies screened) to 100% (13 true positives from 2,037,824 babies screened). It was not possible to calculate sensitivity, specificity, or NPV as there was no systematic follow-up of babies who had screened negative. In a pilot or national screening program for a rare disease using a “promising” test, negative tests will inevitably represent the vast majority of test results. While some studies provided very high PPV, PPV is not intrinsic to the test itself, and at any particular values of sensitivity and specificity, the estimates of PPV (and also NPV) are strongly dependent on disease prevalence. This relationship is illustrated in Supplement 7 over a range of prevalence values similar to those in the included studies and over a range of specificity values. In order to provide a complete assessment of test accuracy, all four metrics (sensitivity, specificity, NPV, PPV) are required.

Whether newborn screening for LCHAD/MTP deficiencies with acylcarnitines measurement in dried blood spots using TMS is appropriate is currently unclear due to a lack of data on babies who screen negative and a lack of consistency between screening test methods. Partial verification bias is a key issue in the included studies; from nearly 4,000,000 babies screened, only 63 (those who screened positive) received a reference standard. Therefore, we cannot know the true disease state of the babies who screened negative. Partial verification bias is common in studies of test accuracy because it is often impractical, unethical, and not cost-effective to follow-up every participant. Alternative approaches to whole population follow-up include statistical methods to attempt to correct for the bias, follow-up of samples of participants who screen negative, and searching disease registers to find false negatives. Statistical methods may introduce other forms of bias (27, 28).

There were substantial differences between screening test methods. For example, there were differences in the extraction and calibration methods (in-house or commercially available test kits); analysis as acylcarnitine butyl esters or free acids (underivatised); screening markers used (e.g., C14OH, C14:1, C16OH, C16:1OH, C18OH, C18:1, C18:1OH, C16OH/C16 ratio); whether markers were employed in isolation or in combination with each other [two studies did not report which marker(s) were used (18, 25)] and variability in the cut-offs between studies, with cut-offs not specified in three studies (18, 23, 25). The majority of FP were found in studies which used lower thresholds for C16OH (>0.03 to >0.10 μmol/L), C16:1OH (>0.04 to 0.15 μmol/L), and C18:1OH (>0.01 to 0.06 μmol/L).

While screening for LCHADD/MTP is conducted in the newborn bloodspot programs of a number of countries, there is little published data on the benefits and harms of these. Taylor-Phillips et al. (29) reviewed the evidence on national policy recommendations on screening newborn babies for rare diseases. They highlight three elements that might determine the balance of benefits and harms from screening programs: test accuracy, the benefit of early detection and treatment, and overdiagnosis (the detection and subsequent treatment of disease that would never have caused symptoms within a person's lifetime). Many of the national policy recommendations (including for LCHADD) did not assess all of these three elements. In relation to screening for LCHADD/MTP, the current review suggests that the evidence on test accuracy is uncertain. A recent systematic review has examined the potential benefit of early detection and treatment, comparing the health outcomes of people with LCHADD/MTP who were treated with pre-symptomatic dietary management following screen detection of the conditions compared with people detected following symptomatic presentation (30). There was some evidence of an association between timing of intervention and outcomes, such as mortality, heart problems, liver problems, visual problems, motor/muscular problems, and hypoglycemia. However, the majority of included studies found no statistically significant differences in outcomes between the two groups. Furthermore, the review identified few studies from which to draw conclusions and high risks of bias in included studies. There is no published evidence on overdiagnosis. Overall, the paucity of data and variability between studies lead to considerable uncertainty regarding the benefits and harms of screening for LCHADD/MTP.

Our review has a number of limitations. First, we were not able to synthesize (meta-analyze) our results numerically due to a lack of data on FN and TN and because of variability between screening test methods. Second, we tailored the QUADAS-2 to reflect newborn screening in the UK; this resulted in high concerns regarding applicability in the patient selection domain, as screening is often conducted sooner after birth in other countries. The definition of a high applicability concern is likely to differ in other countries.

There is currently insufficient evidence to clearly judge test accuracy. This is driven, in part, by a wide range of markers and thresholds being used in the included studies: PPV estimates differed greatly by study, with some suggesting good PPV, albeit on small numbers of cases. It was not possible for us to combine data from different studies or determine which combination of markers and thresholds may yield good accuracy as results were not presented by marker. Future research could involve collaboration between researchers to report scores on a range of relevant markers for cases of LCHAD, cases of MTP, and in the general population using consistent units. There is a precedent for this approach in the form of the Region 4 Stork (R4S) project and accompanying multivariate pattern recognition software (subsequently developed into the interactive web tool Collaborative Laboratory Integrated Reports (CLIR), https://clir.mayo.edu/). The R4S project aimed “(a) to achieve uniformity of testing panels by MS/MS to maximize detection of affected newborns within the region; (b) to improve overall analytical performance; and (c) to set and sustain the lowest achievable rates of false positive and false negative results” (31). Reference and disease ranges for LCHAD/MTP markers were reported for the R4S project (31). To date, the CLIR tool has been used in a small number of research projects (32–35). An additional piece of work should aim to clarify the disease states of babies who screen negative. This could be achieved in a number of ways, such as searching hospital/primary care records or disease registers, or following up samples of babies who have screened negative.

## Conclusions

Measurement of acylcarnitines in newborn dried blood spots using TMS may prove to be a useful way to screen for LCHAD/MTP deficiencies, but currently, there are significant concerns regarding the high number of false positives in some of the studies, risks of bias in the studies, heterogeneity in the methods used, and a lack of data on sensitivity, specificity, or negative predictive values. Clinicians interested in the identification of LCHAD/MTP may consider partnership development across clinical and research networks to address the knowledge gaps identified from this study, including data available for long-term follow-up studies and alignment of diagnostic methodologies.

## Data Availability Statement

The original contributions presented in the study are included in the article/Supplementary Material, further inquiries can be directed to the corresponding author.

## Author Contributions

CS undertook project planning and research design, coordinated the review process, conducted all aspects of the review, and co-wrote the paper with HF. HF undertook project planning, undertook all sifting, sorting, data extraction, and quality appraisal, and co-wrote the paper with CS. JG contributed to sifting and sorting and commented on draft and final versions of the paper. RJ contributed to sifting and sorting and quality appraisal and commented on first and final drafts of the paper. MC undertook project planning, and commented on first and final drafts of the paper. SJ developed and conducted the literature searches, managed references, and helped obtain full-text references. AC undertook project planning, oversight of search strategies and methods, and commented on first and final drafts of the paper. ST-P undertook project planning and research design and commented on first and final drafts of the paper. All members of the team contributed to the development of the protocol. All authors contributed to the article and approved the submitted version.

## Conflict of Interest

The authors declare that the research was conducted in the absence of any commercial or financial relationships that could be construed as a potential conflict of interest.

## Abbreviations

C14OH: 3-hydroxytetradecanoylcarnitine
C14:1: tetradecenoylcarnitine
C16OH: 3-hydroxypalmitoylcarnitine
C16OH/C16: 3-hydroxypalmitoylcarnitine/palmitoylcarnitine
C16:1OH: 3-hydroxypalmitoleylcarnitine
C18OH: 3-hydroxystearoylcarnitine
C18:1OH: 3-hydroxyoleoylcarnitine
C18:1: oleoylcarnitine
DBS: dried blood spot
FP: false positive
LCHAD: long-chain 3-hydroxyacyl-CoA dehydrogenase
MTP: mitochondrial trifunctional protein
NPV: negative predictive value
PPV: positive predictive value
QUADAS-2: Quality Assessment Tool for Diagnostic Accuracy Studies 2
TMS: tandem mass spectrometry
TP: true positive.
