# Supplementary material for: Newborn Screening for Long-Chain 3-Hydroxyacyl-CoA Dehydrogenase and Mitochondrial Trifunctional Protein Deficiencies Using Acylcarnitines Measurement in Dried Blood Spots—A Systematic Review of Test Accuracy
Source: Front Pediatr. 2021 Mar 19;9:606194. doi: 10.3389/fped.2021.606194 (PMC8017228; doi:10.3389/fped.2021.606194)
Supplement: Supplementary file 1 [file Table_1.DOCX]

**Supplement 1.** Search strategies

Search strategy for MEDLINE, MEDLINE In-Process, MEDLINE Daily, Epub Ahead of Print and Embase before deduplication

| Term Group | # | Search terms | Results |
| --- | --- | --- | --- |
| Disease area | 1 | (mitochondrial trifunctional protein adj3 deficien*).ti,ab,kf | 163 |
| Disease area | 2 | mtp deficien*.ti,ab,kf. | 137 |
| Disease area | 3 | trifunctional protein deficien*.ti,ab,kf. | 169 |
| Disease area | 4 | (LCHAD or LCHADD).mp | 522 |
| Disease area | 5 | long chain 3 hydroxyacyl coenzyme A dehydrogenase/ | 198 |
| Disease area | 6 | 3 hydroxyacyl coenzyme A dehydrogenase/ | 1538 |
| Disease area | 7 | HADH Deficien*.mp. | 7 |
| Disease area | 8 | (Hydroxacyl and dehydrogenase).mp. | 15 |
| Disease area | 9 | long chain.mp. | 61746 |
| Disease area | 10 | 8 and 9 | 3 |
| Disease area | 11 | hydroxydicarboxylic acidur*.mp. | 36 |
| Disease area | 12 | Lipid Metabolism, Inborn Errors/ | 2776 |
| Disease area | 13 | fatty acid oxidation disorder*.mp. | 773 |
| Disease area | 14 | Acyl-CoA Dehydrogenase/df [Deficiency] | 2381 |
| Disease area | 15 | Metabolism, Inborn Errors/ | 10175 |
| Disease area | 16 | "inborn errors of metabolism".mp. | 7278 |
| Disease area | 17 | “inborn metabolic disorder*”.mp. | 212 |
| Disease area | 18 | Inherited metabolic disease*.mp. | 2013 |
| Disease area | 19 | 1 or 2 or 3 or 4 or 5 or 8 or 9 or 10 or 11 or 12 or 13 or 16 or 17 or 18 | 35126 |
| Other | 20 | neonatal screening.mp. or exp Neonatal Screening/ | 10526 |
| Other | 21 | neonat* screening.mp. | 10051 |
| Other | 22 | newborn screening.mp | 21485 |
| Other | 23 | ((neonat* or newborn*) adj5 screen*). mp. | 39700 |
| Other | 24 | 20 or 21 or 22 or 23 | 39700 |
| Other | 25 | exp Mass Spectrometry/ or exp Tandem Mass Spectrometry/ or tandem mass spectrometry.mp. | 650977 |
| Other | 26 | mass spectro*.mp. | 715714 |
| Other | 27 | tms.mp. | 25891 |
| Other | 28 | 25 or 26 or 27 | 777839 |
| Other | 29 | 24 and 28 | 2915 |
| Other | 30 | exp dried blood spot testing/ | 6823 |
| Other | 31 | (blood spot* or dry blood spot*).mp. | 17619 |
| Other | 32 | 30 or 31 | 30068 |
| Other | 33 | 29 or 32 | 31873 |
| Other | 34 | 19 and 33 | 1418 |

Search strategy for the Cochrane Library Databases (Searched via the Wiley Online platform)

| Term Group | # | Search terms | Results |
| --- | --- | --- | --- |
| Disease area | 1 | MTP deficien* | 6 |
| Disease area | 2 | Trifunctional protein near/3 deficien* | 7 |
| Disease area | 3 | Lchad or lchadd | 9 |
| Disease area | 4 | MeSH descriptor: [Long-Chain-3-Hydroxyacyl-CoA Dehydrogenase] explode all trees | 1 |
| Disease area | 5 | MeSH descriptor: [3-Hydroxyacyl CoA Dehydrogenases] explode all trees | 86 |
| Disease area | 6 | (Hydroxacyl and dehydrogenase) and long chain | 0 |
| Disease area | 7 | Hydroxydicarboxylic acidur* | 1 |
| Disease area | 8 | MeSH descriptor: [Acyl-CoA Dehydrogenase] explode all trees | 13 |
| Disease area | 9 | MeSH descriptor [Acyl-CoA Dehydrogenase, Long-Chain] explode all trees | 2 |
| Disease area | 10 | Mitochondrial trifunctional protein near/3 deficien* | 0 |
| Disease area | 11 | MeSH descriptor: [Lipid Metabolism, Inborn Errors] this term only | 30 |
| Disease area | 12 | Fatty acid oxidation disorder* | 122 |
| Disease area | 13 | MeSH descriptor: [metabolism, Inborn Errors] this term only | 83 |
| Disease area | 14 | “inborn errors of metabolism” | 206 |
| Disease area | 15 | “inborn metabolic disorder” | 1 |
| Disease area | 16 | “Inherited metabolic disease*” | 317 |
| Disease area | 17 | #1 or #2 or #3 or #4 or #5 or #6 or #7 or #8 or #9 or #10 or #11 or #12 or #13 or #14 or #15 or #16 | 671 |
| Other | 18 | MeSH descriptor: [Neonatal Screening] explode all trees | 302 |
| Other | 19 | Neonat* screening | 1706 |
| Other | 20 | Newborn screening | 1606 |
| Other | 21 | ((newborn* or neonat*) near/5 screen*) | 882 |
| Other | 22 | #18 or #19 or #20 or #21 | 2238 |
| Other | 23 | MeSH descriptor: [Mass Spectrometry] explode all trees | 1493 |
| Other | 24 | MeSH descriptor [Tandem Mass Spectrometry] explode all trees | 619 |
| Other | 25 | Tandem mass spectrometry | 1982 |
| Other | 26 | Mass spectro* | 5420 |
| Other | 27 | Tms | 1468 |
| Other | 28 | #23 or #24 or #25 or #26 or #27 | 4215 |
| Other | 29 | #22 and #28 | 47 |
| Other | 30 | MeSH descriptor: [Dried Blood Spot Testing] explode all trees | 619 |
| Other | 31 | Blood spot* or dreid blood spot* or dry blood spot* | 1331 |
| Other | 32 | #30 or #31 | 1331 |
| Other | 33 | #29 or #32 | 1368 |
| Other | 34 | #17 or #33 | 46 |

Search strategy for Web of Science

| Term Group | # | Search terms | Results |
| --- | --- | --- | --- |
| Disease area | 1 | ((("fatty acid oxidation disorder*" OR "LCHADD" OR "LCHAD" OR "Long-Chain-3-Hydroxyacyl-CoA Dehydrogenase" OR "HADH deficien*" OR ("hydroxyacyl" AND "dehydrogenase" AND "long chain")))) OR TS=(("hydroxydicarboxylic acidur*" or ("inborn errors" and "lipid metabolism"))) OR TS=("mtp deficien*" or ("mitochondrial protein" NEAR/3 deficien*) or ("trifunctional protein" near/3 deficien*)) OR TS =(“inborn errors of metabolism” or “inborn metabolic disorder*” or “inherited metabolic disease*”) OR TS=(“inborn error*” NEAR/3metabolism) | 5739 |
| Other | 2 | TS=(“dry blood spot” or “dried blood spot*” or dbs) | 15660 |
| Other | 3 | TS=((“tandem mass spectro*” or tms or “mass spectro*”)) AND TS=(((newborn* or neonat*) near/5 screen*)) | 1280 |
| Disease area | 4 | #3 OR #2 | 16324 |
| Disease area | 5 | #4 AND #1 | 446 |
