# Supplementary material for: Newborn Screening for Long-Chain 3-Hydroxyacyl-CoA Dehydrogenase and Mitochondrial Trifunctional Protein Deficiencies Using Acylcarnitines Measurement in Dried Blood Spots—A Systematic Review of Test Accuracy
Source: Front Pediatr. 2021 Mar 19;9:606194. doi: 10.3389/fped.2021.606194 (PMC8017228; doi:10.3389/fped.2021.606194)
Supplement: Supplementary file 2 [file Table_2.DOCX]

**Supplement 2.** Adjusted QUADAS-2

*First author surname and year of publication:*

*Name of first reviewer: Name of second reviewer:*

**Phase 1: State the review question:**

3. What is the test accuracy (sensitivity, specificity, and predictive values applicable to UK prevalence) of acylcarnitines measurement in dried blood spots (DBS) using TMS for LCHAD/MTP deficiency screening?

| *Patients (setting, intended use of index test, presentation, prior testing):* |
| --- |
| *Index test(s):* |
| *Reference standard and target condition:* |

**Phase 2: Draw a flow diagram for the primary study**

**Phase 3: Risk of bias and applicability judgments**

*QUADAS-2 is structured so that 4 key domains are each rated in terms of the risk of bias and the concern regarding applicability to the research question (as defined above). Each key domain has a set of signalling questions to help reach the judgments regarding bias and applicability.*

| **DOMAIN 1: PATIENT SELECTION**   1. **Risk of Bias** | |
| --- | --- |
| Describe methods of patient selection: | |
| + Was a consecutive or random sample of patients enrolled? | Yes/No/Unclear |
| + Was a case-control design avoided? | Yes/No/Unclear |
| + Did the study avoid inappropriate exclusions? | Yes/No/Unclear |
| + Were screening samples taken in asymptomatic babies? | Yes/No/Unclear |
| **Could the selection of patients have introduced bias?** | **RISK: LOW/HIGH/UNCLEAR** |
| 1. **Concerns regarding applicability** | |
| Describe included patients (prior testing, presentation, intended use of index test and setting)**:** | |
| **Is there concern that the included patients do not match** **the review question?** | **CONCERN: LOW/HIGH/UNCLEAR** |

| **DOMAIN 2: INDEX TEST(S)**  **If more than one index test was used, please complete for each test.**   1. **Risk of Bias** | |
| --- | --- |
| Describe the index test and how it was conducted and interpreted: | |
| + Were the index test results interpreted without knowledge   of the results of the reference standard? | Yes/No/Unclear |
| + If a threshold was used, was it pre-specified? | Yes/No/Unclear |
| **Could the conduct or interpretation of the index test have introduced bias?** | **RISK: LOW/HIGH/UNCLEAR** |
| 1. **Concerns regarding applicability** | |
| **Is there concern that the index test, its conduct, or interpretation differ from the review question?** | **CONCERN: LOW/HIGH/UNCLEAR** |

| **DOMAIN 3: REFERENCE STANDARD**   1. **Risk of Bias** | |
| --- | --- |
| Describe the reference standard and how it was conducted and interpreted: | |
| + Is the reference standard likely to correctly classify the   target condition? | Yes/No/Unclear |
| + Were the reference standard results interpreted without   knowledge of the results of the index test? | Yes/No/Unclear |
| **Could the reference standard, its conduct, or its interpretation have introduced bias?** | **RISK: LOW/HIGH/UNCLEAR** |
| 1. **Concerns regarding applicability** | |
| **Is there concern that the target condition as defined by the reference standard does not match the review question?** | **CONCERN: LOW/HIGH/UNCLEAR** |

| **DOMAIN 4: FLOW AND TIMING**   1. **Risk of Bias** | |
| --- | --- |
| Describe any patients who did not receive the index test(s) and/or reference standard or who were excluded from the 2x2 table (refer to flow diagram): | |
| Describe the time interval and any intervention between index tests(s) and reference standard: | |
| + Was there an appropriate interval between index test(s) and reference standard? | Yes/No/Unclear |
| + Did all patients receive a reference standard? | Yes/No/Unclear |
| + Did all patients receive the same reference standard? | Yes/No/Unclear |
| + Were all patients included in the analysis? | Yes/No/Unclear |
| **Could the patient flow have introduced bias?** | **RISK: LOW/HIGH/UNCLEAR** |
