# Supplementary material for: Newborn Screening for Long-Chain 3-Hydroxyacyl-CoA Dehydrogenase and Mitochondrial Trifunctional Protein Deficiencies Using Acylcarnitines Measurement in Dried Blood Spots—A Systematic Review of Test Accuracy
Source: Front Pediatr. 2021 Mar 19;9:606194. doi: 10.3389/fped.2021.606194 (PMC8017228; doi:10.3389/fped.2021.606194)
Supplement: Supplementary file 3 [file Table_3.DOCX]

**Supplement 3.** Adjusted QUADAS-2 and guidance notes for LCHADD and MTP

Risk of bias should only be classed as low for each domain if all questions could be answered with ‘yes’. If one or more signaling question is answered with ‘no’ the risk of bias should be classed as ‘high’ and equally if at least one question is answered with ‘unclear’ due to incomplete reporting the risk of bias should be judged ‘unclear’.

**Domain 1: Patient selection**

Acylcarnitine levels in symptomatic older children might be different to levels in asymptomatic patients in the newborn period (Cavedon et al, 2005). Therefore choice of patient population in terms of disease status is an important factor for consideration for bias and applicability.

A. Risk of bias

Guidance:

*Was a consecutive or random sample of patients enrolled?*

This question should only be answered with ‘yes’ if the study clearly states that newborn babies (rather than samples) were recruited consecutively or randomly.

*Was a case-control design avoided?*

We would at least expect a prospective cohort design or the follow up of an existing, pilot or expanded screening programme. Therefore, if the study is a case-control study this question should be answered with ‘No’.

*Did the study avoid inappropriate exclusions?*

If the study excludes >10% of participants with or without specifying reasons, the exclusions should be considered as inappropriate. This cut-off has been determined pragmatically. If studies reporting experiences of implementing screening have a reported screening uptake of at least 95% this question can be answered with ‘yes’.

*Were screening samples taken in asymptomatic babies?*

This question can be answered with ‘yes’ if all samples were taken in babies without clinical symptoms of LCHADD/MTP or were not deemed high risk. The question should be answered with ‘no’ if symptomatic children were tested and the risk of bias classed as ‘high’.

B. Concerns regarding applicability

Guidance:

The timing of sample collection might influence test performance. As the research question aims to address screening of newborns within NHS schedules of 5-8 days, sampling outside this window, e.g. at 2 - 3 days (many European countries and the USA) might not produce outcomes applicable to the NHS context. Studies show mixed results but suggest carnitines C16, C16:1, C18, C18:1, and C18:OH may vary with age at testing so countries not testing round 5-8 days may need differing thresholds (de Tanima et al 2011; Cavedon et al 2005).

Likewise, a cohort study in a country with significantly different prevalence of cases will affect applicability.

Applicability concerns should be regarded ‘high’ if >10% of blood spot specimens were collected in babies >10 days or < 5 days.

Applicability concerns should be also considered ‘high’ if LCHADD prevalence in cohort studies differs significantly from that expected in UK newborns (0.67 per 100,000 [Moorthie et al, 2013).

**Domain 2: Index test**

The main sources of bias introduced by conducting and interpreting the index test are blinding and defining the threshold. If the reference standard is carried out before the index test (e.g. in case control studies) it is important to blind personnel to the results of the reference standard.

The QUADAS-2 tool requires a threshold to be pre-specified in the methods in order to avoid adjustment of the threshold according to the test outcome.

1. Risk of bias

*Were the index test results interpreted without knowledge of the results of the reference standard?*

In case-control design studies blinding needs to be specifically mentioned for this question to be answered with ‘yes’.

*Was a threshold explicitly pre-specified?*

For this question to be answered with ‘yes’ the study needs to mention what kind of threshold was to be used (e.g. Acylcarnitines C16OH ≥0.12, C18-10H, C18OH -) and clearly state that it was specified before the start of the study. If the study reports adjustment to the threshold and reports results according to adjusted thresholds this question should be answered with ‘no’.

B. Concerns about applicability

If the study uses different screening tests to TMS measurement of C16-OH, C18-OH or C18:1-OH as primary markers concerns regarding the applicability of the study should be classed as ‘high’.

**Domain 3: Reference standard**

The most useful test for diagnosis of LCHADD/MTP is measurement of blood, urine and DNA for mutation analysis, which is the method most likely to be adopted in the UK system.{ENBS 2013}. Enzyme analysis in cultured fibroblasts or lymphocytes may also be carried out.

The UK could not currently support extended mutation analysis to determine to test for the specific gene mutation (commonly G1528C IJlst et al, 1996).

Children with LCHADD/MTP can present symptomatically up to childhood, with recorded new cases round age 10 (Kang et al, 2018; Gillingham et al, 2017), so to know whether a true negative case is really a false negative, follow up should be for up to 10 years.

Furthermore, blinding is an issue in studies reporting experiences as only samples with high acylcarnitine samples will be referred to further testing. If the index test is carried out before the reference standard, blinding to the results of the index tests is important.

1. Risk of bias

*Is the reference standard likely to correctly classify the target condition?*

For studies that used urine organic acids, blood acylcarnitine profiles, enzyme analysis in cultured fibroblasts or lymphocytes, or mutation analysis or at least 10 year follow up as reference standard on their own or in combination this question should be answered with ‘yes’.

*Were the reference standard results interpreted without knowledge of the results of the index test?*

This question should be answered with ‘no’ if the study reports an experience report. In case-control studies blinding of the index test results is not an issue and can therefore be classed as ‘yes’. In cohort studies blinding should be specifically mentioned for the question to be answered with ‘yes’.

B. Concerns about applicability

The concern of applicability of the reference standard will be ‘low’ if acylcarnitine levels in blood, urine or molecular genetic testing or at least 10 year follow up were the predefined reference standard in the studies assuming that they all identify LCHADD/MTP and no other type of FAOD.

**Domain 4: Flow and Timing**

Storage of acylcarnitines can be affected by prolonged storage. Up to 330 days at -18 degree centigrade. If stored at room temperatures for >14 days they are hydrolyzed (Fingerhut et al, 2009).

Since LCHADD/MTP is a progressive condition, the time between TMS screening and the reference standard is of importance. Partial and differential verification bias are a concern in studies where screen-negative children are not referred for further testing and receive no reference standard (experience reports) or are followed up for a certain time period only (cohort studies) as these studies have limited data on false negatives.

Children with negative TMS result should be followed up for at least ten years to confirm absence of LCHADD/MTP {Kang et al, 2018; Gillingham et al, 2017} and losses to follow-up should be reported.

1. Risk of bias

*Was there an appropriate interval between index test(s) and reference standard?*

This question can be answered with ‘yes’ if the reference standard is urine organic acids, blood acylcarnitine profiles, enzyme analysis in cultured fibroblasts or lymphocytes, or mutation analysis and the time interval is <15 days. It is very likely that bias will exist if the index test and reference standard (both measuring the same acylcarnitines) are not undertaken at the same time. However, no evidence was identified to base this cut-off on. The question should be answered with ‘no’ if treatment commenced between index test and references standard that decreases the level of acylcarnitines and bias should be regarded as ‘high’. The question can be answered with ‘yes’ if extended mutation analysis or follow-up were used as the reference standard.

*Did all patients receive a reference standard?*

In prospective cohort studies, this question can be answered with ‘yes’ if all those who screen positive have received one of the above mentioned reference standards and those who screened negative were followed up for at least ten years and losses to follow-up are reported and are <10%.

The question should be answered with ‘unclear’ if the study provides no information on how healthy controls were identified in case-control studies and risk of bias should be classed as ‘high’.

Risk of bias should be considered as ‘high’ in case-control studies which included healthy controls identified on the basis of newborn screening results and/or follow-up for less than two years; or in prospective cohort studies which followed up screen negatives for less than two years.

Risk of bias should be considered as ‘high’ in studies that did not specify and describe the reference standard.

*Did all patients receive the same reference standard?*

This question should be answered with ‘no’ if patients received different reference standards or if positive cases received a different reference standard to negative subjects. This question should also be answered with ‘no’ if a list of reference standards is given but no report is made of which patients received which reference standard(s).

If this question is answered with ‘no’, the risk of bias should be regarded as ‘high’ as the different reference standards cannot be classed as equivalent.

*Were all patients included in the analysis?*

If inconclusive or intermediate results are not considered in the analysis the question should be answered with ‘no’. If patients lost to follow up were not included in the analysis or >50% of patients were lost to follow up (even if considered in the analysis) the question should be answered with ‘no’. (The actual proportion of patients lost to follow up needs to be recorded for each study.) If studies report a clinical experience and base test accuracy estimates on interim results and not all patients were followed up the question should be answered with ‘no’. In all three cases the risk of bias should be classed as ‘high’.
