# Supplementary material for: Newborn Screening for Long-Chain 3-Hydroxyacyl-CoA Dehydrogenase and Mitochondrial Trifunctional Protein Deficiencies Using Acylcarnitines Measurement in Dried Blood Spots—A Systematic Review of Test Accuracy
Source: Front Pediatr. 2021 Mar 19;9:606194. doi: 10.3389/fped.2021.606194 (PMC8017228; doi:10.3389/fped.2021.606194)
Supplement: Supplementary file 4 [file Table_4.DOCX]

**Supplement 4.** Publications excluded after review of full-text articles

| No. | Reference | Reason for exclusion |
| --- | --- | --- |
| 1 | Abdel-Hamid, M.;Tisocki, K.; Sharaf, L.;Ramadan, D. 2007. Development, validation and application of tandem mass spectrometry for screening of inborn metabolic disorders in Kuwaiti infants. Medical Principles & Practice, 16(3):215-21 | No data on sensitivity, specificity, PPV or NPV |
| 2 | Alfadhel, M; Al Othaim, A; Al Saif, S; Al Mutairi, F; Alsayed, M; Rahbeeni, Z; Alzaidan, H; Alowain, M;  Al-Hassnan, Z; Saeedi, M; Aljohery, S; Alasmari, A; Faqeih, E; Alwakeel, M; AlMashary, M;  Almohameed, S; Alzahrani, M; Migdad, A; Al-Dirbashi, O. Y; Rashed, M; Alamoudi, M; Jacob, M; Alahaidib, L; El-Badaoui, F; Saadallah, A; Alsulaiman, A; Eyaid, W; Al-Odaib, A. 2017. Expanded Newborn Screening Program in Saudi Arabia: Incidence of screened disorders. Journal of Paediatrics & Child Health, 53: 585-591. | No LCHADD screened |
| 3 | Alratrout, R; Alsadah, Z; Ansari, N. 2017. The frequency of inherited metabolic and endocrine disorders in the eastern and north-western Jawf provinces of Saudi Arabia: Four years data from the newborn screening department, ministry of health, Dammam, Current Pediatric Research. 21 (4), 665-673. | Didn't screen for LCHADD |
| 4 | Cantu-Reyna, C; Zepeda, L. M; Montemayor, R; Benavides, S; Gonzalez, H. J; Vazquez-Cantu, M;  Cruz-Camin, H. 2016. Incidence of inborn errors of metabolism by expanded newborn screening in a Mexican hospital. Journal of Inborn Errors of Metabolism and Screening, 4, 1-8. | LCHADD screened but no cases |
| 5 | Chong, S. C; Law, L. K; Hui, J; Lai, C. Y; Leung, T. Y; Yuen, Y. P. 2017. Expanded newborn metabolic screening programme in Hong Kong: a three-year journey. Hong Kong Medical Journal, 23(5):489-96. | Screened but no cases found |
| 6 | Chrastina, P.; St'astna, S.; Myskova, H.; Kosarova, M.; Elleder, M.; Zeman, J. 2005. Newborn screening of inherited metabolic disorders by tandem mass spectrometry. [Czech]. Klinicka Biochemie a Metabolismus, 13 (2):77-80. | No data on sensitivity, specificity, PPV or NPV |
| 7 | Estrella, J., Wilcken, B., Carpenter, K., Bhattacharya, K., Tchan, M. and Wiley, V. 2014. Expanded newborn screening in New South Wales: missed cases. Journal of Inherited Metabolic Disease, 37(6):881-887. | No data on sensitivity, specificity, PPV or NPV |
| 8 | Feuchtbaum, L; Lorey, F; Faulkner, L; Sherwin, J; Currier, R; Bhandal, A; Cunningham, G. 2006. California's experience implementing a pilot newborn supplemental screening program using tandem mass spectrometry. Pediatrics, 117(5 Pt 2):S261-9. | No data on sensitivity, specificity, PPV or NPV |
| 9 | Filiano, J. J; Bellimer, S. G; Kunz, P. L. 2002. Tandem mass spectrometry and newborn screening: pilot data and review. Pediatric Neurology, 26 (3), 201–204. | Cost paper and did not include LCHADD |
| 10 | Fleischman, A; Thompson, J D; Glass, M. 2014. Systematic Data Collection to Inform Policy Decisions: Integration of the Region 4 Stork (R4S) Collaborative Newborn Screening Database to Improve MS/MS Newborn Screening in Washington State, Jimd Reports, 23(3), 201-204. | Not LCHADD |
| 11 | Guo, K; Zhou, X; Chen, X; Wu, Y; Liu, C; Kong, Q. 2018. Expanded newborn screening for inborn errors of metabolism and genetic characteristics in a Chinese population. Frontiers in Genetics, 20(9), 122. | Didn't screen for LCHADD |
| 12 | Hannon, H; Lim, T; Adam, B; Therrell, B. 2003. Outcomes from tandem mass spectrometry (MS/MS) workshops in the United States and the performance evaluation of MS/MS laboratories. Southeast Asian Journal of Tropical Medicine & Public Health, 34(3), 121-126. | Not population testing |
| 13 | Harms, E; Olgemoller, B. 2011, Neonatal Screening for Metabolic and Endocrine Disorders. Deutsches Arzteblatt International, 108(1-2):11-21. | Not test accuracy |
| 14 | Hassan, F. A.; El-Mougy, F.; Sharaf, S. A.; Mandour, I.; Morgan, M. F.; Selim, L. A.; Hassan, S. A.; Salem, F.; Oraby, A.;Girgis, M. Y.; Mahmoud, I. G.; El-Badawy, A.; El-Nekhely, I.; Moharam, N.; Mehaney, D. A.; Elmonem, M. A. 2016, Inborn errors of metabolism detectable by tandem mass spectrometry in Egypt: The first newborn screening pilot study. Journal of Medical Screening, 23(3):124-129. | Not test accuracy |
| 15 | Huang, H. P; Chu, K. L; Chien, Y. H; Wei, M. L; Wu, S. T; Wang, S. F; Hwu, W. L. 2006. Tandem mass neonatal screening in Taiwan--report from one center. Journal of the Formosan Medical Association, 105 (11), 882-886. | Didn't test for LCHADD |
| 16 | Johnson, A. W; Mills, K; Clayton, P. T. 1996. The use of automated electrospray ionization tandem MS for the diagnosis of inborn errors of metabolism from dried blood spots. Biochemical Society Transactions, 4(3), 932-938. | Conference abstract |
| 17 | Kasper, D.C., Ratschmann, R., Metz, T.F., Mechtler, T.P., Moslinger, D., Konstantopoulou, V., Item, C.B., Pollak, A. & Herkner, K.R. 2010 The national Austrian newborn screening program - eight years’ experience with mass spectrometry. Past, present, and future goals. Wiener Klinische Wochenschrift, 122, 607-13. | No data on sensitivity, specificity, PPV or NPV |
| 18 | la Marca, G; Malvagia, S; Casetta, B; Pasquini, E; Donati, M. A; Zammarchi, E. 2008. Progress in expanded newborn screening for metabolic conditions by LC-MS/MS in Tuscany: update on methods to reduce false tests. Journal of Inherited Metabolic Disease, 31(S2):S395-404. | No cases |
| 19 | Lee, H. C; Mak, C. M; Lam, C. W; Yuen, Y. P; Chan, A. O; Shek, C. C; Siu, T. S; Lai, C. K; Ching, C. K;  Siu, W. K; Chen, S. P; Law, C. Y; Tai, H. L; Tam, S; Chan, A. Y. 2011. Analysis of inborn errors of metabolism: disease spectrum for expanded newborn screening in Hong Kong. Chinese Medical Journal, 124(7):983-989. | No cases and not clear that they screened for LCHADD |
| 20 | Lim, J.S., Tan, E.S., John, C.M., Poh, S., Yeo, S.J., Ang, J.S., Adakalaisamy, P., Rozalli, R.A., Hart, C., Tan, E.T., Ranieri, E., Rajadurai, V.S., Cleary, M.A. & Goh, D.L. 2014. Inborn Error of Metabolism (IEM) screening in Singapore by electrospray ionization-tandem mass spectrometry (ESI/MS/MS): An 8 year journey from pilot to current program. Molecular Genetics & Metabolism, 113, 53-61. | No data on sensitivity, specificity, PPV or NPV |
| 21 | Naylor, E. W; Chace, D. H. 1999. Automated tandem mass spectrometry for mass newborn screening for disorders in fatty acid, organic acid, and amino acid metabolism. Journal of Child Neurology, 14 (1), S4-S8. | Didn’t screen for LCHADD |
| 22 | Niu. D.M., Chien, Y.H., Chiang, C.C., Ho, H.C., Hwu, W.L., Kao, S.M., Chiang, S.H., Kao, C.H., Liu, T.T., Chiang, H & Hsiao, K.J. 2010. Nationwide survey of extended newborn screening by tandem mass spectrometry in Taiwan. Journal of Inherited Metabolic Disease, 33, S295-305. | No data on sensitivity, specificity, PPV or NPV |
| 23 | Scolamiero, E; Cozzolino, C; Albano, L; Ansalone, A; Caterino, M; Corbo, G; di Girolamo, M. G; Di Stefano, C; Durante, A; Franzese, G; Franzese, I; Gallo, G; Giliberti, P; Ingenito, L; Ippolito, G; Malamisura, B; Mazzeo, P; Norma, A; Ombrone, D; Parenti, G; Pellecchia, S; Pecce, R; Pierucci, I; Romanelli, R; Rossi, A; Siano, M; Stoduto, T; Villani, G. R; Andria, G; Salvatore, F; Frisso, G; Ruoppolo, M. 2015. Targeted metabolomics in the expanded newborn screening for inborn errors of metabolism, Molecular Biosystems, 11(6):1525-1535. | No mention of screening for LCHADD |
| 24 | Shibata, N; Hasegawa, Y; Yamada, K; Kobayashi, H; Purevsuren, J; Yang, Y; Dung, V. C; Khanh, N. N; Verma, I. C; Bijarnia-Mahay, S; Lee, D. H; Niu, D. M; Hoffmann, G. F; Shigematsu, Y; Fukao, T; Fukuda, S;  Taketani, T; Yamaguchi, S. 2018. Diversity in the incidence and spectrum of organic acidemias, fatty acid oxidation disorders, and amino acid disorders in Asian countries: Selective screening vs. expanded newborn screening. Molecular Genetics and Metabolism Reports, 16, 5-10. | No data on sensitivity, specificity, PPV or NPV |
| 25 | Tal, G; Pitt, J; Morrisy, S; Tzanakos, N; Boneh, A. 2015. An audit of newborn screening procedure: impact on infants presenting clinically before results are available. Molecular Genetics & Metabolism, 114(3), 403-408. | Didn't screen for LCHADD |
| 26 | Vilarinho, L.; Rocha, H; Sousa, C.;Marcao, A; Fonseca, H; Bogas, M; Osorio, R. V. 2010. Four years of expanded newborn screening in Portugal with tandem mass spectrometry. Journal of Inherited Metabolic Disease, 33(supp3):S133-8. | Not test accuracy |
| 27 | Wilcken, B; Wiley, V; Hammond, J; Carpenter, K. 2003. Screening newborns for inborn errors of metabolism by tandem mass spectrometry, New England Medical Journal, 348(23):2304-12. | No cases |
| 28 | Yunus, Z. M; Rahman, S. A; Choy, Y. S; Keng, W. T; Ngu, L. H. 2016. Pilot study of newborn screening of inborn error of metabolism using tandem mass spectrometry in Malaysia: outcome and challenges. Journal of Pediatric Endocrinology & Metabolism, 29(9):1031-1039. | No data on sensitivity, specificity, PPV or NPV |
