# Supplementary material for: Newborn Screening for Long-Chain 3-Hydroxyacyl-CoA Dehydrogenase and Mitochondrial Trifunctional Protein Deficiencies Using Acylcarnitines Measurement in Dried Blood Spots—A Systematic Review of Test Accuracy
Source: Front Pediatr. 2021 Mar 19;9:606194. doi: 10.3389/fped.2021.606194 (PMC8017228; doi:10.3389/fped.2021.606194)

**Supplement 5.** Risk of bias and applicability concerns summary: review authors' judgements about each domain for each included study.


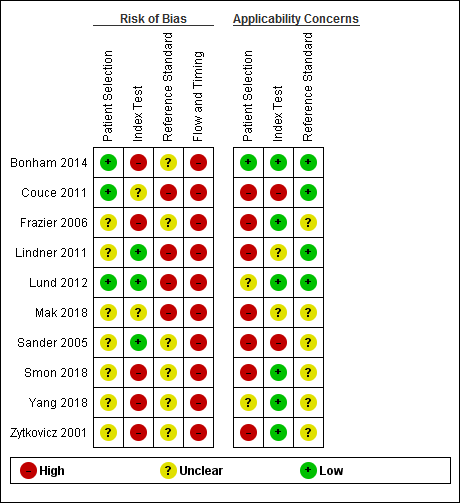

Supplement: Supplementary file 5 [file Table_5.DOCX]
