# Supplementary material for: Newborn Screening for Long-Chain 3-Hydroxyacyl-CoA Dehydrogenase and Mitochondrial Trifunctional Protein Deficiencies Using Acylcarnitines Measurement in Dried Blood Spots—A Systematic Review of Test Accuracy
Source: Front Pediatr. 2021 Mar 19;9:606194. doi: 10.3389/fped.2021.606194 (PMC8017228; doi:10.3389/fped.2021.606194)
Supplement: Supplementary file 6 [file Table_6.DOCX]

Supplement 6. Study characteristics of included studies

| **Study reference** | **Country, time period** | **Study design** | **Source and type of material** | **Age at specimen collection** | **Method of extraction and derivatisation** | **Type of TMS and conditions** | **Analytes and cut-off** | **Re-testing of positive samples and reference standard** |
| --- | --- | --- | --- | --- | --- | --- | --- | --- |
| **ASIA** | | | | | | | | |
| Mak 2018 | Hong Kong  1 October 2012-31 August 2014 | Pilot screening study in 2,440 neonates.  3 public hospitals and the University of Hong Kong | Heel prick test in compliance with Clinical and Laboratory Standard Institute Guidance. | 24h-28 days  *(24 - 48h n=2064 [84.6%],*  *3 - 5 days n=331 [13.6%],*  *5 - 7 days n=9 [0.4%],*  *7 - 28 days n=36 [1.5%]).* | Two commercial DBS assay kits:  (1) MassChrom® Amino Acids and Acylcarnitines from Dried Blood/Non Derivatised (Chromsystems Instruments & Chemicals GmbH, Germany); and  (2) NeoBase^TM^ Non-derivatized MSMS kit (PerkinElmer, USA).  Fully automated online extraction system  (DBS-MS 500; CAMAG, Muttenz, Switzerland).  (1) and (2) No derivatisation. | LC-TMS.  Acylcarnitines: MRM. No further details provided | Not specified which markers were used for LCHADD/MTPD | Positive samples reanalysed.  Reference standard: measurements of functional metabolites (mainly plasma acylcarnitine levels, and urine organic acid levels) and genetic diagnosis by DNA sequencing wherever appropriate. |
| Yang 2018 | China  2014 – 2015  (1 year) | Propsective newborn screening study in 100,077 neonates.  Jining city.  Number of centres not reported | DBS on Whatman 903 paper. | 3 to 37 days | NeoBase^TM^ Non-derivatized MSMS kit (Perkin Elmer, MA, USA).  No derivatisation. | Triple quadrupole TMS equipped with an ACQUITY TQ detector (Waters, MA, USA) coupled to a Waters 1525 binary HPLC pump.  MRM mode. | LCHADD: C16OH  >0.04 µmol/L  MTPD: C18OH  >0.03 µmol/L  C18:1OH >0.05 µmol/L | TMS test repeated and compared to first results.  Urinary organic acids determined by GC-MS, or high precision DNA mass spectrometry for 39 genetic mutations. |
| **EUROPE** | | | | | | | | |
| Bonham 2014 | United Kingdom  July 2012 – July 2013  (1 year) | Prospective expanded newborn screening study in 436,969 neonates.  6 screening laboratories. | DBS on Guthrie filter paper. | 5-8 days as per UK regulations | Extraction not reported.  Underivatised eluates. | MRM TMS  *m/z* 416 to 82 transition. | Analytical cut-off:  C16OH  >0.12 μmol/L.  Screening cut-off:  C16OH  >0.15 μmol/L – lowered from 0.20 μmol/L following a missed case.  C16:1OH; C18OH.  Cut-off NR. | If analytical cutoff for C16OH positive, assay C16OH, C16:1OH and  C18OH in duplicate (using fresh disks) and use screening cutoff.  Referred directly to specialist metabolic physician.  Reference standard: blood acyl carnitines, urine organic acids, blood DNA G1528C mutation analysis, and enzyme assay if needed. |
| Couce 2011 | Galicia, Spain  July 2000 – July 2010  (10 years) | Prospective newborn screening study in 210,165 neonates. | DBS on Whatman 903 paper. | Up to 2002:  5-8 days.  2003-2010: on day 3. | NR | MS/MS Applied Biosystems Sciex 2000 apparatus | C16OH,  C18:1OH, C18OH  Cut-offs not reported | When a positive result was found in the DBS sample, repeat or additional analyses were performed on the urine samples.  Clearly aberrant results immediately referred to clinical unit.  For borderline results, a second sample was requested by NBS lab. If second result also positive, the patient was referred to clinical unit.  Reference test: Enzyme and/or molecular studies. |
| Lindner 2011  Schulze 2003 | South-West Germany  April 1998- September 2001 {Schulze 2003}  January 1999 - June 2009  (10 years)  {Lindner 2011} | Prospective newborn screening programme in 250,000 neonates up to 2001. Overlap with 1,084,195 screend newborns reported by Lindner et al. (2011).  1 screening laboratory. | DBS on Schleicher & Schuell 2992 up to 1999, then on Schleicher & Schüll 903 since 2000. | Up to 2001:  Recommended time between 3rd – 7th days of life. Median 5^th^ day. {Schulze 2003}    Up to 2005: Recommended between day 3-5.  2005 onwards:  36-72 hours.{Lindner 2011} | Extraction using methanol stock solution of internal deuterated standards (0.04 µmol/L [^2^H_9_]myristoylcarnitine [C14], 0.08 µmol/L [^2^H_3_]palmitoylcarnitine [C16]).  Derivatisation to butyl esters.  When a specific stable isotope was not available, the following ratios were used for calculation:  C14OH/C14-d9, C16:1/C16-d3, C16:1OH/C16-d3, C16OH/C16-d3, C18:1/C16-d3, C18/C16-d3, and C18:1OH/C16-d3. | API 365 triple quadruple TMS with an ion spray device.  Acylcarnitines measured by positive precursor ion scan of *m/z* 85 (scan range *m/z*: 200–500). | C14OH (>0.12 µmol/L) and/or C16:1OH (>0.22 µmol/L); C16OH (>0.20 µmol/L); C18:1OH (>0.12 µmol/L); C18OH (>0.11 µmol/L).  Cut-off prespecified: 99.5^th^ percentile based on data collected from 10,000 healthy neonates. | Repeat analysis from the same blood spot: only TP if both samples positive. If a distinct discrepancy, a third test was done and the mean of the 2 corresponding results was used and interpreted by an experienced metabolic disease specialist.  Presumptive positive cases repeated DBS test on recall or referral to a treatment centre.  Reference standard: Enzyme activity in fibroblasts/lymphocytes {Schulze 2003} – acylcarnitine profile in plasma/DBS and/or genotype and/or enzyme activity {Lindner 2011}. |
| Lund 2012 | Denmark, Faroe Islands and Greenland  Trial period: February 2002 – February 2009  (7 years).  Routine expanded screening: February 2009 – March 2011. | Prospective pilot and routine expanded screening programme in 504,049 newborns.  1 screening laboratory. | DBS on Schleicher & Schüll 903 filter paper up to 2010, then replaced by Ahlstrom 226 filter paper. | Trial period:  Recommended age 4-9 days after birth (median 5 days).  From February 2009:  Recommended age 2-3 days; median age 2.5 days. | February 1st, 2002, to June 30th, 2003:  Analysis using a protocol developed at Statens Serum Institut, Copenhagen based on analysis of relevant acylcarnitine butyl esters.  From July 1st, 2003,  until February 2nd, 2009:  PerkinElmer NeoGram Amino acids and acylcarnitines tandem mass spectrometry kit™ (MS-8970EY).  Derivatisation to butyl esters.  From February 2nd, 2009: PerkinElmer NeoBase non-derivatized MSMS kit™ (3040-0010).  No derivatisation. | SciEx API2000 up to 30^th^ June 2003; acylcarnitines as precursors of *m/z* 85.  TMS conditions unclear for time period July 2003-February 2009.  From 2^nd^ February 2009:  Waters Micromass Quattro micro™ tandem mass  spectrometers. | Primary: C16OH >0.12U;  Secondary: C18:1OH >0.1U.  Cut-off pre-specified. | Positive samples reanalaysed in duplicates.  If abnormal profiles confirmed, the Centre for IMD Copenhagen University Hospital contacted for confirmatory tests.  Reference standard: Urine organic acids, plasma acylcarnitines, molecular-genetic analyses. |
| Sander 2005 | Germany  1999 – 2005  (6 years) | Prospective screening programme in 1,200,000 neonates.  1 screening laboratory. | DBS on S&S 2992 filter paper (Schleicher & Schüll). | Recommended age:  36-72 hours.  97.5% by day 5. Some several days later. | Extraction using methanol containing deuterated internal standards (D_3_-C14, D_3_-C16 and D_3_-C18) and derivatisation to butyl esters. | 3 TMS (TMS quatro LC, Micromass, Manchester).  Long-chain hydroxyacylcarnitines measured in MRM mode; remaining acylcarnitines and free carnitine in full scan MCA mode. | C14:1  >0.35 µmol/L;  C14OH  >0.2 µmol/L;  C16OH  >0.08 µmol/L;  C18:1OH >0.06 µmol/L  Threshold chosen after evaluation of data from patients with known MTPD and 5,000 normal controls. | Repeated analyses of fresh dried blood spots.  Reference standard: Enzyme analysis in cultured fibroblasts or lymphocytes, mutation analysis. |
| Smon 2018 | Slovenia  2013-2014 | Retrospective pilot study of blood spots in 10,048 newborns. | DBS. | 48-72 hours.  Analysed after 6-11 months storage (unclear how stored). | Chromsystems’ kit “Amino Acids and Acylcarnitines from Dried Blood” (Graefelfing, Germany):  Analytes were extracted using the extraction buffer with added internal standards.  Derivatisation to butyl esters. | PerkinElmer 200 HPLC system coupled to AB Sciex 3200 QTRAP (AB Sciex, Singapore). | C16:1OH, C16OH, C18:1OH, C16OH/C16  Cut-offs set at 99.9^th^ percentiles after the completion of the study. | Children with the highest probability of IEM were invited for an outpatient visit at the University Children’s Hospital for follow-up tests.  Reference standard: Next-generation sequencing, organic acids in urine, additional acylcarnitine test using DBS. |
| **USA** | | | | | | | | |
| Frazier 2006 | North Carolina, USA  July 1997 – July 2005  (8 years). | Prospective newborn screening programme in 944,078 neonates.  2 screening laboratories. | DBS on Schleicher & Schüll 903 filter paper. | At least 24 hours regardless of gestational age and feeding status.  Mean age 39 hours. | July 1997 - April 1999:  Performed at Neo Gen laboratory using commercial testing.  From April 1999:  In-house extraction method by Millington et al. (1990) {REF}:  Extraction using methanol with deuterated internal standards.  Derivatisation to butyl esters. | Micromass Ltd/Water Corp Quattro LC TMS  Gilson instruments 215 Liquid Handler autosampler and Hewlett Packard/Agilent Technologies series 1100 Isocratic HPLC pump. | Since January 2003:  C16OH  >0.18 µmol/L (single cut-off);  C18:1  >4.08 µmol/L (independent elevation not diagnostic),  C18:1OH >0.14 µmol/L (independent elevation not diagnostic).  Cut-offs initially set at ~4 SD above the mean of results obtained from 2,000 sequential newborn samples.  Cut-offs were modified over time to minimise FP and FN. | Repeat analysis from another disk punched from the same card.  If results were borderline a request was sent for a repeat sample. If above the diagnostic cut-off referal to a metabolic specialist.  Reference standard: Urine organic acids and a plasma acylcarnitine profile.  Enzyme and mutation analyses done whenever the tests were available and approved by 3^rd^ party reimbursers. |
| Zytkovicz 2001 | New England Newborn Screening Program (NENP) testing newborns in Massachusetts, but also from Maine, New Hampshire, Vermont, and Rhode Island (USA).  1999-2001  (2 years) | Prospective screening programme in 257,000 neonates.  Only 164,000 Massachusetts newborns were screened for LCHADD.  1 screening laboratory. | DBS on S&S Grade 903 filter paper (Schleicher & Schüll). | Most DBS obtained 1-3 days after birth. | In-house method:  Extraction using methanolic internal standard solution ([^2^H_3_]-palmitoylcarnitine  ([^2^H_3_]-C16), 0.06 µmol/L).  Derivatisation to butyl estesr. | 1100 Hewlett Packard HPLC pump.  Model 215 Gilson autosampler sent sample to MS/MS.  Micromass Quattro LC triple-quadrupole TMS.  Analyzed with MRM mode; for acylcarnitines (35–54 V, 25 eV) ion abundance of the fragment ions was determined at *m/z* 85. | C16OH/d-C16  Cut-off: 0.1.  Cut-off set at 7 SD above population mean such that ~0.02% or less of population of ~4,000 random newborns would be flagged for each marker. | Repeat analysis from another disk punched from the same card.  Mean of the 2 tests was calculated, and if it was greater than the flag value, results were reported to follow up.  If one of more markers were out of range a full metabolic work up was recommended.  If initial specimen had very increased marker then full metabolic work up initiated straight away.  Reference standard: According to standard metabolic criteria. |

DBS, dried blood spots; DNA, deoxyribonucleic acid; FN, false negative; FP, false positive; GC, gas chromatography; HPLC, high-performance liquid chromatography; IEM, inborn errors of metabolism; IMD, inherited metabolic disease; LC, liquid chromatography; LCHADD, long-chain 3-hydroxyacyl-CoA dehydrogenase deficiency; MCA, multi-channel acquisition; MRM, multiple reaction monitoring; MTPD, mitochondrial trifunctional protein deficiency; NBS, newborn blood spot screening; NR, not reported; SD, standard deviation; TMS, tandem mass spectrometry; TP, true positive.
