# Supplementary material for: Newborn Screening for Long-Chain 3-Hydroxyacyl-CoA Dehydrogenase and Mitochondrial Trifunctional Protein Deficiencies Using Acylcarnitines Measurement in Dried Blood Spots—A Systematic Review of Test Accuracy
Source: Front Pediatr. 2021 Mar 19;9:606194. doi: 10.3389/fped.2021.606194 (PMC8017228; doi:10.3389/fped.2021.606194)
Supplement: Supplementary file 7 [file Table_7.DOCX]

**Supplement 7.** Example positive predictive values for a test with sensitivity of 0.9990 and specificity of 0.999990 – 0.999999, calculated for a disease with a prevalence of 0.0% - 0. 000007%
